# Bioactive Compounds from Plant-Based Functional Foods: A Promising Choice for the Prevention and Management of Hyperuricemia

**DOI:** 10.3390/foods9080973

**Published:** 2020-07-23

**Authors:** Lin-Lin Jiang, Xue Gong, Ming-Yue Ji, Cong-Cong Wang, Jian-Hua Wang, Min-Hui Li

**Affiliations:** 1 Department of Pharmacy, Inner Mongolia Medical University, Hohhot 010110, China; jianglinlin27@163.com; 2 Department of Pharmacy, Baotou Medical College, Baotou 014060, China; gongxue_2017@yeah.net (X.G.); Jimingyue9@163.com (M.-Y.J.); WangCongCong0@163.com (C.-C.W.); 3 Department of Pharmacy, Qiqihar Medical University, Qiqihar 161006, China; 4 Pharmaceutical Laboratory, Inner Mongolia Institute of Traditional Chinese Medicine, Hohhot 010020, China; 5 Inner Mongolia Key Laboratory of Characteristic Geoherbs Resources Protection and Utilization, Baotou Medical College, Baotou 014060, China

**Keywords:** hyperuricemia, plant-based functional food, xanthine oxidase, adenosine deaminase, uric acid transporter, bioactive compound

## Abstract

Hyperuricemia is a common metabolic disease that is caused by high serum uric acid levels. It is considered to be closely associated with the development of many chronic diseases, such as obesity, hypertension, hyperlipemia, diabetes, and cardiovascular disorders. While pharmaceutical drugs have been shown to exhibit serious side effects, and bioactive compounds from plant-based functional foods have been demonstrated to be active in the treatment of hyperuricemia with only minimal side effects. Indeed, previous reports have revealed the significant impact of bioactive compounds from plant-based functional foods on hyperuricemia. This review focuses on plant-based functional foods that exhibit a hypouricemic function and discusses the different bioactive compounds and their pharmacological effects. More specifically, the bioactive compounds of plant-based functional foods are divided into six categories, namely flavonoids, phenolic acids, alkaloids, saponins, polysaccharides, and others. In addition, the mechanism by which these bioactive compounds exhibit a hypouricemic effect is summarized into three classes, namely the inhibition of uric acid production, improved renal uric acid elimination, and improved intestinal uric acid secretion. Overall, this current and comprehensive review examines the use of bioactive compounds from plant-based functional foods as natural remedies for the management of hyperuricemia.

## 1. Introduction

Hyperuricemia (HUA) is a common metabolic disease caused by an imbalance between endogenous production and excretion of urate [1]. Recently, considerable evidence has indicated that uric acid (UA), an endogenous antioxidant present in low concentrations in the human plasma, plays an active role in life processes [2,3]. However, the over-production of UA easily results in the formation of monosodium urate crystals, which increases inflammation and causes gout [4]. In recent years, the incidence of HUA has been increasing every year worldwide, and the prevalence of HUA is particularly high in China and the United States [5,6,7]. HUA is considered to be a major risk factor of metabolic disorders after hypertension, hyperlipidemia, and hyperglycemia [8], and is considered to be the major pathological basis of gout, whereby approximately 5–12% of HUA patients have the possibility of developing gout [9]. Moreover, a large number of epidemiological studies have reported that HUA is closely related to diabetes, hypertension, obesity, cardiovascular disease, and kidney disease [10,11,12,13], which suggests that complications associated with HUA may increase in the coming years.

**Figure 1:**
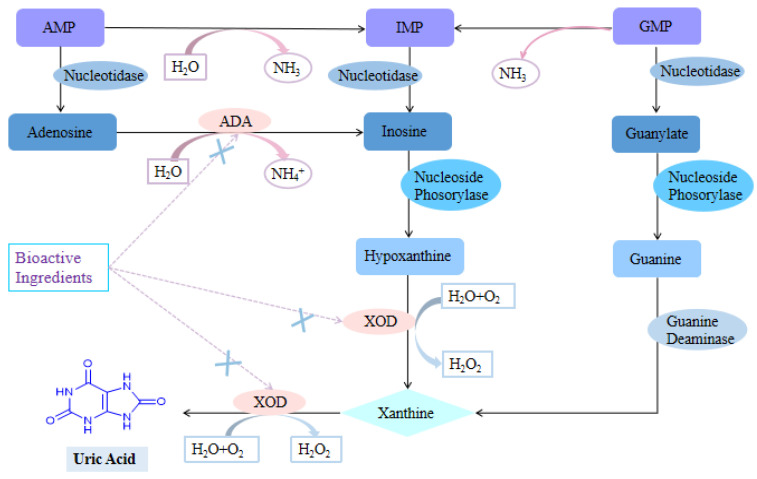
Xanthine oxidase (XOD) and adenosine deaminase (ADA) inhibitory mechanisms of bioactive ingredients.

**Figure 2:**
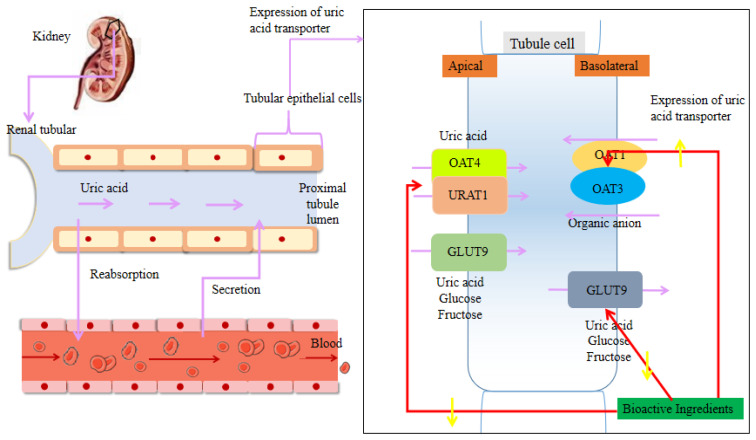
Effect of bioactive components of plant-based functional foods on renal transporters.

As mentioned above, HUA can be caused by either an increase in UA production or a decrease in UA metabolism in the body, with reduced urate excretion being the most common mechanism, accounting for about 90% of cases [9]. As a result, a means to reduce the body UA levels could be considered an effective treatment. However, the production and metabolism of UA are complex physiological processes. Endogenous UA is derived from the metabolism of nucleic acids within the body, and accounts for 80–90% of the body’s total UA content [14,15]. Xanthine oxidase (XOD) and adenosine deaminase (ADA) are key enzymes that catalyze the production of UA. XOD is known to catalyze the oxidation of hypoxanthine to xanthine and xanthine to UA [16], while also converting purines from protein-rich foods into UA. In addition, ADA catalyzes the conversion of adenosine to inosine, which in turn is catalyzed to hypoxanthine and xanthine. Therefore, ADA plays a key role in indirectly catalyzing the formation of UA [17]. The catalytic process for UA production is presented in Figure 1. In contrast, exogenous UA is derived from the intake of purine-containing foods. As previously reported, the consumption of seafood, animal giblets, eggs, soy products, wheat, sugary beverages, and high-fructose foods will increase UA production, which is associated with a high risk of gout and HUA [18,19,20]. During UA metabolism, approximately 70% of UA is eliminated through kidneys and the other 30% by the intestinal pathway [21]. In humans, UA metabolism in the body mainly takes place in the kidneys, involving processes such as reabsorption and secretion (Figure 2). UA relies on the cooperative excretion of multiple transporters to complete metabolism, whereby the urate transporter 1 (URAT1), the organic anion transporters 4 (OAT4), and the glucose transporter 9 (GLUT9) mainly regulate UA reabsorption, while the organic anion transporters 1 (OAT1) and 3 (OAT3) are responsible for regulating renal UA excretion [22,23,24,25]. Therefore, lowering UA levels can be achieved by inhibiting UA synthesis and promoting UA metabolism, as well as encouraging high-risk people to change their dietary structure can prevent and control HUA. Currently, the clinical drugs available for HUA can be categorized into UA synthesis inhibitors (allopurinol, febuxostat, etc.) and UA excretion promoters (probenecid, benzbromarone, etc.) [26]. Although these drugs aid in reducing UA levels, many exhibit serious side effects, such as gastrointestinal reactions, skin rashes, liver and kidney dysfunction, and hepatotoxicity [27]. It is therefore necessary to discover alternative effective agents for the treatment of HUA.

Plant-based functional foods are derived from natural or unprocessed plant foods, or plant foods modified via biotechnological means [28]. They are products that have a relevant effect on well-being and health or reduce the risk of disease [29]. Interestingly, many such functional foods have been links with lowered incidences of various health disorders, such as cardiovascular disease, diabetes, cancers, and gout, and so there is growing interest in the research and development of plant-based functional foods [30,31,32,33]. In recent years, with the increase in numbers of HUA patients, studies into the treatment of HUA using plant-based functional foods have received increasing attention. For example, sea buckthorn was found to exhibit high antioxidant capacity and XOD inhibition capacity, while lemon water extract can directly promote the metabolism of excess UA, thereby indicating a potential to treat HUA [34,35,36]. Previous studies have found that these functions are associated with the presence of large quantities of phytochemicals, which are chemical compounds originating from plants [37]. Furthermore, bioactive components from plant-based functional foods have been extensively screened for their potential anti-HUA activities both in vivo and in vitro [32,38]. Table 1 briefly summarizes the animal models of HUA. The identified constituents can be divided into six categories, namely flavonoids, phenolic acids, saponins, alkaloids, polysaccharides, and others. This review highlights the biological components of plant-based functional foods towards the treatment of HUA, as well as the mechanisms by which these components exhibit hypouricemic effects. We expect that the contents of this review will aid in the understanding of potential applications for plant-based functional foods in the treatment of HUA.

## 2. Bioactive Components of Plant-Based Functional Foods

Flavonoids are polyphenols with a basic 2-phenyl-chromone structure [39], and they are found widely in plants. Hence, flavonoids are introduced to the human diet through vegetables, fruits, grains, tea, and other plant-derived foods [40]. Previous studies have demonstrated that they are also known to be potent inhibitors of XOD and ADA, and they could significantly reduce the production of UA [41]. Indeed, molecular docking results indicate that the hydrophobic action of flavonoids plays an important role in the binding of such compounds to XOD, and various tested flavonoids are competitive inhibitors. More specifically, the hydroxyl groups at the C-7 and C-5 positions, in addition to the carbonyl group at the C-4 position, interact with a large number of XOD amino acid residues, which promotes hydrogen bonding and electrostatic interactions with XOD [42,43]. Upon increasing the affinity between flavonoids and XOD, a stronger XOD inhibition ability was achieved, whereby substitution at the C-7 position of the basic flavonoid structure was particularly effective [40]. In addition, flavonoids have also been found to promote UA excretion through regulation of the UA transporters in the kidneys, such as URAT1. The molecular virtual docking study has revealed that the hydroxyl group of morusin can combine with the oxygen in the structure of URAT1 to form a hydrogen bond, resulting in an inhibitory effect on the expression of URAT1, which is superior to that of the known drug benzbromarone [44].

At present, only a few clinical studies have shown that flavonoids from plant-based functional foods can effectively lower UA levels. For example, following the isolation of puerarin from *Pueraria lobata* (Willd.) Ohwi and Wang et al. randomly divided 120 HUA patients into a control group, a myricetin group, and a puerarin group. After injection with 5 mL/d puerarin injection and 5 mL/d myricetin, the changes in the UA levels in patients with HUA were observed. The obtained results showed that the serum uric acid (SUA) levels of the myricetin and puerarin groups decreased significantly (*p* < 0.05), indicating that myricetin and puerarin present obvious therapeutic effects on HUA [45].

Quercetin, one of the major flavonols mainly found in onions and sophora japonica (*Sophora japonica* L.), exhibits a variety of biological activities. In their study into the effect of quercetin on HUA rats, whereby a rat model of HUA was induced via the administration of potassium oxonate. Xie et al. found that after three weeks of administration, quercetin (10 mg/kg/d) significantly reduced the levels of SUA and inhibited the activities of XOD and ADA in both serum and the liver (*p* < 0.05) [46]. In addition, one clinical trial investigated the effect of oral quercetin over four weeks on the SUA levels in 22 healthy male volunteers with high baseline SUA. It was found that the oral administration of 500 mg/d quercetin significantly lowered plasma UA levels 26.5 μM, while the extraction of UA and the patient blood pressure were not affected [47]. Furthermore, molecular docking studies confirmed that quercetin could bind to the XOD active center, which prevents xanthine from entering the XOD active center and thereby inhibits XOD activity [48]. Quercetin is therefore able to inhibit the catalytic activities of both XOD and ADA to reduce the production of UA.

*Ipomoea batatas* L. possesses a high content of anthocyanins (ACNs) [49]. In one study, ACNs were administered to potassium oxonate, inosine, and yeast-induced HUA mice model for three weeks, and it was found that the groups treated with 400 and 800 mg/kg of ACNs exhibited significant reductions in their SUA levels by 30.2% and 37.9%, respectively (*p* < 0.01), in addition to effective decreases in the serum and liver XOD activities in mice (*p* < 0.05). Furthermore, treatment with high doses of ACNs significantly down-regulated the mRNA expression levels of the URAT1 and GLUT9 (*p* < 0.001), while up-regulating the mRNA expression levels of OAT1, OAT3, and ATP-binding cassette subfamily G member 2 (ABCG2) (*p* < 0.05) in the kidney. Moreover, ACNs treatment lowered blood urea nitrogen (BUN) and serum creatinine (Scr) levels, while up-regulating the mRNA expression levels of organic cation transporters (OCT1 and OCT2) and organic carnitine transporter (OCTN1 and OCTN2) compared with the model group (*p* < 0.01). It has also been suggested that ACNs exhibit hepatoprotective activities and nephroprotective effects, thereby suggesting overall that ACNs are potential treatments for HUA [50].

**Figure 3:**
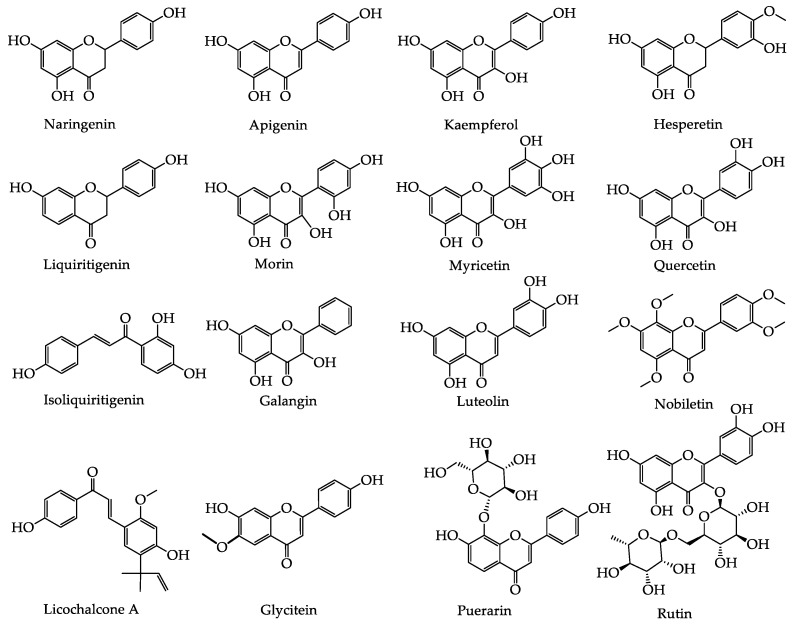
The basic structure of hypouricemic effects from flavonoids bioactive components in plant-based functional foods.

The hypouricemic effect of flavonoids has been studied in particular detail compared to other natural products. Although the majority of previous studies have focused on the inhibition of XOD, the number of studies on UA transporters are gradually increasing. Besides, studies on monomeric compounds remain scarce, and in general, there is a lack of high-quality clinical research. For example, it has been shown that stevia residue extract can reduce SUA levels in HUA mice, and this was attributed to the presence of flavonoids [51], thereby indicating that the effects of flavonoids in the treatment of HUA require further study. The established hypouricemic effects and mechanisms of action of the bioactive components of flavonoids in plant-based functional foods are summarized in Table 2 and Table 3, and the structures of flavonoids obtained from these plant-based functional foods are illustrated in Figure 3.

### 2.1. Phenolic Acids

Phenolic acids, which are secondary metabolites, are non-flavonoid phenolic compounds. They represent a substantial part of the human diet [70]. In recent years, phytochemicals such as phenolic acids have been found to exhibit XOD and ADA inhibitory activities, and are thought to be applicable in the prevention and treatment of HUA. For example, compounds such as chicory acid, caffeic acid, and chlorogenic acid, inhibit the activity of XOD [71].

In the context of chicory acid, which was isolated from *Cichorium intybus* L., Zhu et al. established a quail HUA model to elucidate the active ingredients and mechanism of *C. intybus* L. in combating HUA. After 21 days of administration, chicory acid (150 mg/kg/d) significantly reduced the quail SUA (*p* < 0.05). Moreover, the quail serum ADA and XOD levels were also significantly reduced (*p* < 0.05), which may be related to inhibition of the XOD and ADA activities. Overall, chicory acid significantly reduced quail SUA levels by inhibiting the XOD and ADA activities [72,73].

Phenolic antioxidants, including phenolic acids, have been isolated from Adlay (*Coix lachryma-jobi* L.) [74]. Upon the administration of various doses of caffeic acid (i.e., 25, 50, and 100 mg/kg) to potassium oxonate-induced HUA rats, the UA levels were reduced in the high-dose group (*p* < 0.05). Moreover, the BUN and Scr levels were significantly reduced compared with the model control group, and caffeic acid was found to reduce BUN to the normal range. Besides, an in vivo study showed that caffeic acid regulated the transcription levels in a dose-dependent manner by up-regulating the expression of UA secretory transporters OAT1 and ABCG2 mRNA, and down-regulating UA reabsorption transporters URAT1 and GLUT9 mRNA. Furthermore, an in vitro study showed that caffeic acid can inhibit XOD by competitively binding to xanthine, with an IC_50_ value of 53.45 µM being recorded [75]. Therefore, it is believed that caffeic acid presents a dual effect in lowering UA levels, and so presents a potential application for the treatment of HUA.

**Figure 4:**
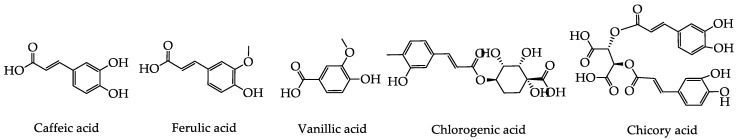
The basic structure of hypouricemic effects from phenolic acid bioactive components in plant-based functional foods.

However, to date, despite extensive research into phenolic acids, few studies exist regarding the regulation of UA transporters. In addition, there is a lack of anti-HUA clinical data, and few reports have been published on the role of phenolic acids in regulating transport proteins. These issues must, therefore, be solved to enhance the applicability of phenolic acids for the treatment of HUA. The hypouricemic effects and mechanisms of action of the various bioactive phenolic acids found in plant-based functional foods are listed in Table 4, and their structures are illustrated in Figure 4.

### 2.2. Alkaloids

Alkaloids are a class of nitrogen-containing organic compounds that exist in many organisms [77]. Due to their complex structures and strong biological activities, their role in lowering UA should not be ignored. Recently, it has been reported that alkaloids can not only inhibit XOD and ADA activities, but also play a role in promoting UA excretion and inhibiting UA reabsorption [78].

Recently, Sang et al. evaluated the effective components present in a total alkaloid extract from *Nelumbinis folium* (lotus leaf) for the lowering of the UA levels. UHPLC-Q-TOF-MS and 3D docking analysis were employed to show that roemerine was a potentially active component. More specifically, roemerine bound with XOD through hydrophobic interactions, inhibited the activity of XOD, and reduced the production of UA [78,79]. Furthermore, nuciferine, a major aporphine alkaloid of the lotus leaf, was found to decrease SUA levels and improve kidney function in potassium oxonate-induced HUA mice. After seven days of treatment, the SUA, BUN, and Scr levels were dramatically reduced (*p* < 0.05), and the excretion of UA increased significantly (*p* < 0.05) for the high-dose nuciferine group (40 mg/kg). It has since been reported that the mechanism of lowering UA is related to down-regulation of the expression of URAT1, GLUT9, and up-regulation of the expression of OAT1 and ABCG2 in HUA mice [80].

Evodiamine is the main active component of *Evodia rutaecarpa* (Juss.) Benth, and has been shown to exhibit an obvious effect on lowering SUA levels. Tao et al. established animal models of HUA in rats and chickens to observe the effect of evodiamine on lowering SUA after 7 and 14 days of administration. Compared with the model group, the low and high dosage groups (9 and 18 mg/kg) reduced SUA values in rats, while the low dose group showed significantly reduced SUA values in HUA chicken [81]. Similarly, Song et al. studied the effects and mechanisms of evodiamine dispersing tablets (5 mg/kg) on SUA in HUA chickens. Their results showed that after 14 days of the administration, UA levels were significantly reduced, as were the activities of XOD and ADA. This experiment suggested that evodiamine dispersible tablets could apply to the treatment of HUA by lowering UA levels in clinical applications [82].

**Figure 5:**
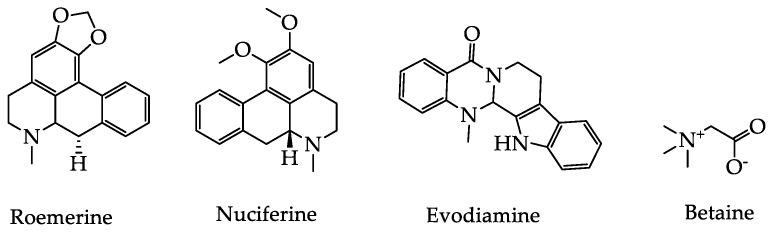
The basic structure of hypouricemic effects from alkaloids bioactive components in plant-based functional foods.

Although alkaloids have been shown to prevent and control HUA through a variety of mechanisms, exhibiting a significant effect on lowering UA levels, due to a lack of clinical data, larger numbers of studies must be conducted in the context of clinical trials and toxic doses. The hypouricemic effects of alkaloid bioactive components in plant-based functional foods and their mechanisms of action are summarized in Table 5, while their structures are illustrated in Figure 5.

### 2.3. Saponins

Saponins are mainly distributed in terrestrial plants, and small amounts are also found in marine life. Based on their different aglycones, saponins can be divided into steroidal saponins and triterpenoid saponins. In recent years, studies have shown that saponins can reduce UA production by inhibiting the activity of XOD and ADA, and that they can also increase UA excretion by regulating the expression of UA transporters [86].

As an example, the anti-HUA mechanism dioscin, which is mainly distributed in *Dioscorea opposita* L. [87], was investigated in HUA mice. More specifically, HUA mice were induced with potassium oxonate (250 mg/kg), and dioscin was orally administered to HUA mice at dosages of 319.22, 638.43, 1276.86 mg/kg/d for 10 days. Following treatment, the SUA levels were significantly reduced, and the Scr levels were lower than those found in the model group (*p* < 0.05). In addition, dioscin significantly increased the 24 h cumulative urinary excretion of creatinine (*p* < 0.05), the protein level of renal mOAT1 in HUA mice treated by dioscin increased significantly at high dosages (1276.86 mg/kg/d), and the protein level of renal mURAT1 decreased. Thus, the mechanism by which dioscin lowers UA levels involves transporter regulation [88].

HUA rats were also treated with saponins from *Gynostemma pentaphyllum* (Thunb.) Makino (GPS). Compared with the model group, the low (15 mg/kg) and high dosage groups (60 mg/kg) exhibited dramatically reduced SUA levels (*p* < 0.01, *p* < 0.05). Moreover, after being treated with a high dosage of GPS, the UA level was close to normal (*p* < 0.01), and the levels of XOD and ADA in the serum and liver decreased (*p* < 0.05). Treatment with GPS also increased the kidney index, downregulated URAT1 and GLUT9 expression, and upregulated OAT1 expression in the kidney. GPS may, therefore, be an effective treatment for HUA through the inhibition of XOD and ADA, and an increase in UA excretion by regulation of the URAT1, GLUT9, and OAT1 transporters [89,90].

Overall, saponin extracts act by inhibiting UA generation, promoting UA excretion, and protecting the kidneys. At present, research into the anti-HUA activities of saponin extracts are in their initial stages, and the identification of additional pharmaceutically active monomer components is necessary. Moreover, investigations into the pharmacological mechanisms of any monomeric species are desirable, as is the development of safe and effective new drugs demonstrating anti-HUA properties.

### 2.4. Polysaccharides

Significant attention has been paid to the extraction and bioactivity of biomacromolecules, such as polysaccharides. Polysaccharides obtained from natural sources tend to exhibit a low toxicity in addition to various bioactivities, such as anti-bacterial, anti-inflammatory activities [91,92]. In recent years, studies reporting their inhibition of UA production and enhancement of UA elimination have been published, indicating that polysaccharides may be a candidate for the development of new natural anti-HUA agents.

*Lonicera japonica* (Thunb) is recognized as a medicine food homology species. The *L. japonica* polysaccharides have been studied for their hypouricemic effect in potassium oxonate-induced HUA mice. Interestingly, with an increase in the polysaccharide dose, the level of UA was significantly lowered, demonstrating that the oral administration of polysaccharides could treat HUA in a dose-dependent manner. In particular, in the high-dose group, the level of SUA was reduced, and compared with the model group, SUA levels in the middle and low dose groups decreased by 47.93% and 43.41%, respectively (*p* < 0.01). The low, middle, and high dose groups (100, 200, and 300 mg/kg) showed the capability to inhibit the activity of XOD (28.71%, 46.31%, and 54.69%, respectively), thereby indicating that *L. japonica* (Thunb) polysaccharides could significantly attenuate HUA in rats [93].

To further study the effect of pachman (polysaccharides of Poria Cocos, PPC) on HUA, Wang et al. fed rats with potassium oxonate and ethambutol to establish an animal model for HUA, and then treated with PPC. The high-dose PPC group (2.0 g/kg/d) exhibited an increase in the fractional excretion of UA, while the level of SUA and any pathological changes in renal tubules were reduced compared with the model group (*p* < 0.05). The protein expression results showed that the expression of URAT1 was significantly down-regulated compared with the model group, while the expression of OAT1 was significantly increased. These results revealed that PPC increased the reabsorption of UA by down-regulating the expression of URAT1, while up-regulating the expression of OAT1 to reduce the re-secretion of UA [94].

Some polysaccharides have also been shown to play an important role in reducing UA. However, previous studies have shown that a high fructose intake was associated with a higher risk of gout and HUA. For example, Lecoultre et al. evaluated 16 healthy adults who were induced by a high fructose, which found UA clearance rate was decreased and SUA was increased [95]. Moreover, an increase in SUA induced by fructose metabolism could have some effects on kidney injury [96]. Cirillo et al. showed that fructose can induce proximal tubular injury in vitro by fructokinase to generate oxidants and UA [97]. As a result, further studies into the pharmacological effects of polysaccharides are required, as are the corresponding clinical trials.

### 2.5. Others

**Figure 6:**
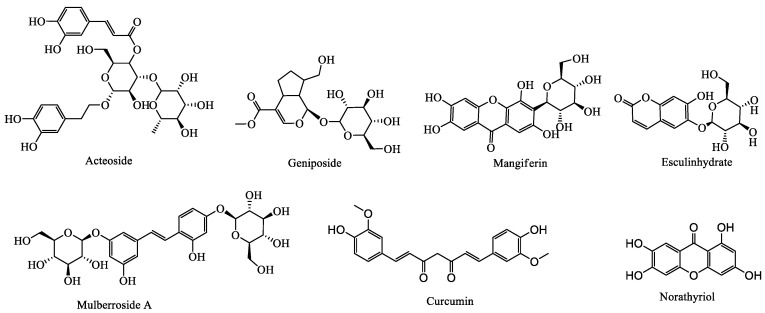
The basic structure of hypouricemic effects from other bioactive components in plant-based functional foods.

**Figure 7:**
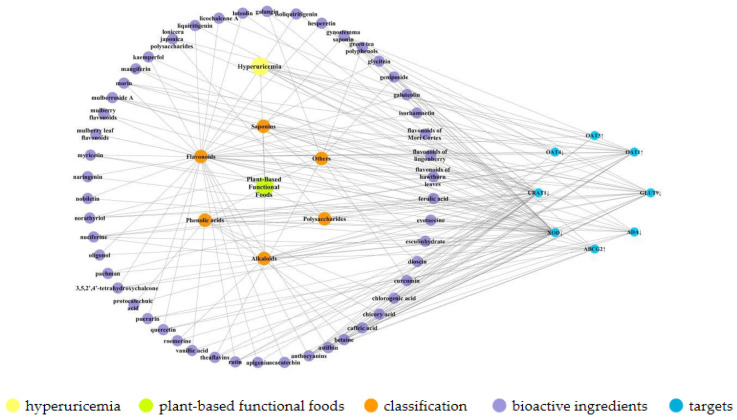
The hypouricemic effects on the compounds–targets network from the plant-based functional foods.

In addition to the above bioactive ingredients, other components (e.g., terpenoids, stilbene glycosides, and coumarin) have also been found to exhibit UA-lowering effects. For example, gardenoside and acteoside have been reported to present significant hypouricemic effects [98,99]. Moreover, Moriwaki et al. studied changes in SUA concentration after intake of oligonol in six healthy subjects. Subjects were treated with 2 g/d oligonol, 1h UA excretion, and partial UA clearance were significantly reduced, with decreased SUA concentration [100]. The UA-lowering effects and mechanisms of action of these other components are summarized in Table 6, and their basic structures are shown in Figure 6. Moreover, compounds-targets network diagrams for the plant-based functional foods exhibiting hypouricemic effects were established using Cytoscape 3.7.1 software, as shown in Figure 7. In Figure 7, the bioactive compounds of plant-based functional foods for hyperuricemia are divided into six categories, namely flavonoids, phenolic acids, alkaloids, saponins, polysaccharides, and others. The compounds-targets results show that XOD is a major target for UA reduction of plant-based functional foods active ingredients. Besides, except for polysaccharides, other plant-based functional foods active ingredients of plants act on CLUT9 targets and all but saponins on URAT1. Moreover, flavonoids, saponins, and phenolic acids can inhibit the activity of ADA to inhibit the production of UA. In addition, saponins, phenolic acids, and polysaccharides can act on OAT1 targets. Furthermore, among these components, only flavonoids could down-regulate OAT4 and up-regulate OAT3 expression.

## 3. Uric Acid Reduction Effects of Plant-Based Functional Foods

As mentioned above, the bioactive components of plant-based functional foods prevent UA disorders by inhibiting the enzyme responsible for UA production, by enhancing the excretion of UA, and by preventing its reabsorption. These mechanisms of action are summarized as follows.

### 3.1. Inhibition of Uric Acid Production

XOD and ADA are key enzymes that catalyze the production of UA. XOD catalyzes the oxidation of hypoxanthine to xanthine and xanthine to UA [16]. ADA plays a key role in indirectly catalyzing the formation of UA [17]. Thus, inhibiting UA synthesis through XOD and ADA inhibition can be considered a therapeutic target to reduce the level of UA in the body. Indeed, in vitro and animal studies have indicated that bioactive components of plant-based functional foods could inhibit XOD and ADA. Notably, increasing molecular docking studies have revealed that polyphenols could bind to an amino acid of XOD and enter the molybdopterin center to form a complex, effectively inhibiting XOD [109]. Further studies have shown that flavonoids and phenolic acids not only exhibit good XOD inhibitory activities, but also present a certain ability to scavenge oxygen free radicals, which can alleviate the damage caused to the body by peroxides generated by XOD [110].

Luteolin is mainly present in *L. japonica* Thunb. and *Dendranthema morifolium* (Ramat.) Tzvel., and exhibits its anti-HUA effect by inhibiting the excess production of UA [111]. Hao et al. showed that the intragastric administration of luteolin (20, 40, and 80 mg/kg) to potassium oxonate-induced HUA mice for seven days reduced the levels of UA and XOD in a dose-dependent manner. Compared with the model group, the high-dose group showed significantly reduced levels of SUA, XOD, BUN, and Scr (*p* < 0.01) [53]. In another study, Yan et al. found that luteolin presents a competitive inhibitory effect on XOD. They postulated that the mechanism of this activity involves luteolin binding to amino acids in the active site of XOD at a single binding site, which is mainly driven by hydrophobic interactions. Molecular docking results revealed that a combination of luteolin and XOD changed the conformation of XOD, and inhibited the synthesis of UA [112], thereby confirming the XOD inhibition activity and hypouricemic effects of luteolin.

Lipid emulsion-induced HUA rats have been used to study the effects of GPS on lowering UA levels. In this study, the low (15 mg/kg) and high dosage groups (60 mg/kg) presented dramatically reduced SUA levels (*p* < 0.01, *p* < 0.05), whereby the UA level was close to normal for the high dosage group (*p* < 0.01). Moreover, the serum and liver levels of XOD and ADA also decreased (*p* < 0.05). These results confirmed that GPS significantly reduced the production of UA through inhibition of the XOD and ADA activities [90].

Galangin extracted from *Rhizoma Alpiniae* was used to treat HUA mice, with high and medium dose groups (300 and 150 mg/kg) resulting in lower UA levels than for the model group (*p* < 0.05), and a significantly decreased XOD activity for the high dose group (*p* < 0.05) [113]. Recently, Zhang et al. reported the XO inhibitory mechanism of galangin, predicting that galangin could enter the Mo center and occupy the catalytic center of XOD to inhibit the activity of XOD, thereby preventing xanthine from entering the active center to block the generation of UA [114].

### 3.2. Regulation of the Renal Uric Acid Transporter

Physiologically, UA production and excretion are in a dynamic balance. However, when UA excretion is reduced and excess UA is produced, thereby leading to HUA [98]. As a result, promoting the excretion of UA could be an effective means to treat HUA. In the process of promoting excretion, UA relies on the cooperative excretion of multiple transporters on the proximal tubular epithelial cells of the apical and basolateral membranes [22]. In addition, various studies have confirmed that the reabsorption of UA can be categorized into two steps, namely UA uptake into renal tubular epithelial cells through the anion transporters *SLC22A12* (URAT1) and *SLC22A11* (OAT4), and release into the blood through the anion transporter SLC2A9 (GLUT9) from the renal tubular epithelial basolateral membrane [23,24,25]. GLUT9 has two splice variants, which are GLUT9a and GLUT9b. GLUT9a of the renal tubular epithelial cell basolateral membranes is responsible for UA reabsorption, while URAT1 and OAT4, localized in the apical membrane of proximal tubules, also control renal urate reabsorption. In contrast, organic anion transporters on the basolateral membrane of renal proximal tubules (e.g., *SLC22A6* (OAT1) and *SLC22A8* (OAT3)), in addition to GLUT9b on the apical membrane of proximal tubules, play an important role in the secretion of UA [22].

Numerous studies have demonstrated that the bioactive components of plant-based functional foods increase UA elimination by up-regulating (OAT1, OAT3) and down-regulating (URAT1, OAT4, and GLUT9) UA transporters in the kidneys [33]. They also improve renal function by regulating the transport and excretion of organic cations (OCTs) and carnitine transporters (OCTNs) in the kidneys [115,116], and so are likely to be important agents in the treatment of HUA. 

Theaflavins are important functional ingredients in black tea. Among them, studies have shown that the three theaflavin monomer, namely theaflavin (TF), theaflavin-3-gallate (TF-3-G), and theaflavin-3-3′-gallate (TFDG) exhibit significant hypouricemic effects on HUA mice. Compared with the model group, the TF (20, 50, and 100 mg/kg), TFDG (50 and 100 mg/kg), and TF-3-G (100 mg/kg) groups notably decreased SUA levels (*p* < 0.01), while TFDG (20 mg/kg) and TF-3-G (50 mg/kg) also clearly reduced the SUA levels (*p* < 0.05). These results indicated that the hypouricemic effect of TF was superior to those of TFDG and TF-3-G at the same dosage. With the exceptions of TFDG (20 mg/kg) and TF-3-G (20 mg/kg), BUN was also reduced in other treatment dose groups (*p* < 0.01), while TF(20 mg/kg), TFDG (20 and 50 mg/kg), and TF-3-G (20, 50, and 100 mg/kg) also decreased Scr (*p* < 0.001). These components could, therefore, treat renal damage in HUA mice by decreasing BUN and Scr levels. In addition, they down-regulated the expression of the genes and proteins of GLUT9 and URAT1, while up-regulating the gene and protein expression of OCTN1, OCT1, OCT2, OAT1, and OAT2 [117]. These findings indicated that TF, TFDG, and TF-3-G could exhibit potential application prospects in the prevention and therapy of HUA.

The effects of licochalcone A from the *Glycyrrhiza uralensis* Fisch were also investigated in a 60 mice model of HUA induced by potassium oxonate and xanthine. The results indicated that licochalcone A can significantly reduce the level of SUA of HUA mice, increase the excretion of UA, and reduce the level of Scr and BUN. Besides, licochalcone A can notably inhibit the transport activity of OAT4 [55]. Besides, a further study has also investigated the effect of green tea polyphenols (GTP) on potassium oxonate-induced HUA mice, and explored the underlying mechanisms of action. It was reported that GTP significantly decreased the SUA levels of HUA mice in a dose-dependent manner (*p* < 0.05), while GTP dosages of 300 and 600 mg/kg markedly reduced the XOD activities in the serum and liver of HUA mice (*p* < 0.05). Furthermore, these dosages reduced the expression of URAT1 (*p* < 0.05), as well as increasing the expression of OAT1 and OAT3 in the kidneys (*p* < 0.01). Overall, the results suggested that GTP reduced UA levels by inhibiting UA production and increasing its excretion [101].

Furthermore, to examine the lowering UA effects of rutin from Sophora japonica (*Sophora japonica* L.), a potassium oxonate-induced HUA model was established in mice. HUA mice were randomly divided into six groups. Compared to that in control mice, treatment with rutin (50, and 100 mg/kg) caused significant reduction SUA, Scr, and BUN, serum and kidney uromodulin levels, while elevating UA excretion in HUA mice. Further, rutin was administered orally 1 h, significantly downregulated mRNA and protein levels of mice GLUT9 and URAT1, and upregulated mRNA and protein levels of OAT1 and OCTs in the kidney of HUA mice. In conclusion, rutin exerted its hypouricemic and renal function improvement by the regulation of renal organic ion transporters [64].

In addition, the SLC2A9 and SLC22A12 are mentioned as genes that have been found to play a role in regulating SUA concentrations through urate reabsorption. However, genetic variants in SLC22A12 and SLC2A9 can result in hereditary renal hypouricemia 1 and 2, leading to severe hypouricemia [118]. In the study, hypouricemia reflected excessive UA excretion, which may lead to UA stones and acute renal failure. In everyday life, people with hypouricemia should eat more foods rich in antioxidants, such as glutathione, vitamin E, vitamin C, etc. At present, vitamin C and E have been isolated in plant-based functional foods. For example, Vitamin C is found in blueberries. Moreover, sea buckthorn berry is rich in vitamins C and E [119,120,121]. Therefore, these plant functional foods may also have the potential to prevent hypouricemia.

### 3.3. Enhancement in Intestinal UA Secretion

Similar to the kidneys, the intestines also play an important role in the excretion of UA. To maintain normal daily body UA levels, approximately two-thirds of UA is eliminated through the kidneys while the other third is eliminated by the intestinal pathway [122]. Urate transport is a complex process involving several transmembrane proteins that promote reabsorption (e.g., URAT1, GLUT9) and secretion (ABCG2). ABCG2 plays a significant role in regulating UA transport in the gastrointestinal tract and is a high-capacity urate transporter that is most active in the jejunum and the ileum [123]. It plays a crucial role in renal urate overload and extra-renal urate underexcretion. ABCG2 dysfunction leads to the blockade of renal and intestinal urate excretion, thereby inducing HUA due to a renal urate overload and its overflow into the kidney. The ABCG2 population-attributable percentage risk for HUA has been reported to be 29.2%, which is significantly higher than those with more typical environmental risks [124]. Stiburkova et al. studied 58 patients with primary HUA and 176 patients with gout in the Czech Republic, among whom 17 patients with HUA and 14 patients with gout were pediatric-onset patients. At the same time, 115 cases of normal anemia control group were compared. Fifteen ABCG2 exons were amplified and sequenced. The chi-square fitting test was used to compare the small allele frequencies, and the logarithmic rank test was employed to compare the empirical distribution functions. The obtained results suggested that genetic factors affecting the ABCG2 function should be considered routinely in the diagnosis of hyperuricemia/gout, especially in pediatric patients [123].

However, the mechanisms involved in the elimination of UA from the intestine remain unclear. To date, only a few studies have shown that ABCG2 is the main UA transporter to maintain serum UA levels, with its most active expression being in the jejunum and the ileum [125]. 

In addition, Morimoto et al. found that the expression of ABCG2 in an HUA rat group was up-regulated in the intestinal villi and crypt. They confirmed that ABCG2 is involved in the intestinal excretion of UA in humans and rats as an extrarenal excretion pathway, thereby providing some clarification regarding UA metabolism along the intestine by focusing on a novel UA exporter, ABCG2 [126]. Furthermore, Wang et al. [127] confirmed that chicory extract ameliorates intestinal UA elimination by modulating the ABCG2 transporter. In 10% fructose-induced HUA rats, the administration of chicory water extract (6.6 g/kg) significantly reduced SUA levels, and significantly increased intestinal UA excretion (*p* < 0.05). Compared with the model group, ABCG2 was up-regulated on the jejunum and the ileum. Further research showed that chicory can significantly increase ABCG2 mRNA expression to reduce UA levels in the jejunum and ileum.

## 4. Conclusions and Future Perspectives

HUA can lead to life-threatening disorders that are rapidly increasing in frequency worldwide, and so the consumption of functional foods could be considered an alternative to medication to prevent or treat HUA. In this context, plant-based functional foods are of particular interest since they contain thousands of naturally beneficial phytochemicals. Numerous in vitro and in vivo experiments have therefore been conducted to elucidate the mechanism by which these plant-based foods lower UA levels, whereby active ingredients such as flavonoids, phenolic acids, and alkaloids reduce the production of UA or promote its excretion. Indeed, it was confirmed that plant-based functional foods are very helpful for the management of UA disorders; however, these studies have their limitations. For example, although animal models can fully reflect the pharmacological actions and metabolic processes of active ingredients, rapid and simple screening is challenging due to the long cycle, and current animal models are limited due to differences in the UA metabolism in humans and animals. In addition, the majority of in vitro experiments carried out to date mainly screen for XOD inhibition, and there is a lack of comprehensive animal model base studies. Furthermore, the anti-HUA effects of many plants have been examined without the determination of the bioactive compounds responsible for their activities, and there is a lack of substantial clinical data and dose-toxicity data to support the applicability of bioactive ingredients in humans. Moreover, there is a lack of relevant research data on intestinal UA elimination by bioactive compounds, and studies focusing on the mechanisms by which such active ingredients act are scarce and vague. 

Novel approaches are therefore required to identify bioactive ingredients from plant-based functional foods, evaluate their efficacy in human and animal models, and develop a sustainable and natural means of treating or preventing HUA. In this context, molecular docking technology has been used to elucidate the mechanism and structural characteristics of polyphenols inhibiting XOD, which is of great significance for the development and synthesis of XOD inhibitors for the treatment of HUA. Since the function of the UA transporter is essential for the maintenance of normal UA levels, further studies into the function of the UA transporter will provide a new strategy for the treatment of HUA and related diseases. In addition, the intestinal tract should be investigated in further detail as a new route to UA secretion, providing a potential new target for the development of natural drugs against HUA. In combination with clinical trials, a comprehensive study into the bioactive ingredients present in plant-based functional foods is necessary, and the diets of patients at high risk from suffering high UA levels should be altered. In conclusion, a large number of studies have confirmed that many biologically active compounds from plant-based functional foods possess anti-HUA activities, which therefore provides a theoretical basis for the synthesis of novel anti-HUA drugs, and suggests the potential of plant-based functional foods for the future prevention and management of HUA.

## Figures and Tables

**Table 1:** Establishment of an animal model of Hyperuricemia (HUA) by drugs.

No.	Drug	Animal	Dosage (mg/kg)	Mode of Administration
A	Potassium oxonate	Mice	-	Intragastric administration
B	Potassium oxonate	Rats	200	Intragastric administration
C	Potassium oxonate	Mice	250	Oral gavage
D	Potassium oxonate	Mice	250	Intragastric administration
E	Potassium oxonate	Mice	270	Intragastric administration
F	Potassium oxonate	Mice	300	Intragastric administration
G	Potassium oxonate	Mice	500	Intragastric administration
H	Adenine	Mice	75	Intragastric administration
I	Adenine + potassium oxonate	Mice	100 + 250	Intragastric administration
J	Adenine + ethylamine butanol	Rats	-	Intragastric administration
K	Inosine + potassium oxonate	Rats	400 + 280	Intragastric administration
L	Yeast + potassium oxonate	Rats	1500 + 200	Intragastric administration
M	Purine	Mice	300	Intragastric administration
N	Uric acid	Rats	150	Intragastric administration
O	Uric acid	Rats	180	Intragastric administration
P	High purine diet	Rats	-	Oral gavage
Q	Yeast	Quails	6 mL	Oral gavage
R	High purine diet	Quails	-	Oral gavage

**Table 2:** Experiment and mechanism of flavonoids bioactive components from plant-based functional foods on hyperuricemia.

Source	Bioactive Compound	Model	Dose	Effects	Mechanisms	Ref.
*Apium graveolens* L./Celery	Apigenin	C	40 and 80 mg/kg	SUA, urinary UA and the protein expression of URAT1 levels were significantly decreased, while 24 h urinary creatinine were significantly increased	This is associated with promoting renal excretion of UA by down-regulating the expression of URAT1	[52]
*Apium graveolens* L./Celery	Kaempferol	E	150 and 300 mg/kg	Significantly decreased SUA	Inhibit UA production by inhibiting XOD	[53]
*Camellia sinensis* var. Assamica/Pu-erh tea	Myricetin	D	4 mg/kg	Significantly lowered SUA level, it also markedly inhibited liver XOD and ADA activities	It is mainly involved in inhibiting UA production by inhibiting XOD and ADA activities	[54]
*Glycyrrhiza uralensis* Fisch/Liquorice Root	Liquiritigenin	G	10 mg/kg	SUA level significantly reduced, fractional excretion of UA was increased	This is related to promoting renal excretion of UA by down-regulating the transport expression of URAT1	[55]
*Glycyrrhiza uralensis* Fisch/Liquorice Root	Isoliquiritigenin	G	10 mg/kg	SUA level significantly reduced, fractional excretion of UA was increased	This is related to inhibiting UA reabsorption by down-regulating the transport of OAT4	[55]
*Glycyrrhiza uralensis* Fisch/Liquorice Root	Licochalcone A	G	10 mg/kg	SUA level significantly reduced, fractional excretion of UA was increased	This is related to inhibiting UA reabsorption by down-regulating the transport of OAT4	[55]
*Vaccinium vitisidaea* L./Lingonberry	Flavonoids from fruit residues of lingonberry	B	100 and 200 mg/kg	SUA was significantly reduced at 100 mg/kg, while 200 mg/kg inhibited the activity of XOD in liver	It is mainly involved in inhibiting XOD activity	[56]
*Smilax china* L./Rhizome Glabrous Greenbrier	Astilbin	B	10 and 20 mg/kg	SUA, Scr, and BUN were significantly reduced, and urinary UA and renal UA excretion effectively increased	It is related to promoting renal excretion of UA by suppressing role in GLUT9 and URAT1 expression and up-regulating the expression of ABCG2, OAT1, OAT3	[57]
*Pueraria lobata* (Willd.) Ohwi/Pueraria	Puerarin	L	200 mg/kg	SUA, and BUN were significantly reduced	It is mainly involved in inhibiting XOD activity to inhibit UA production	[58]
*Glycyrrhiza uralensis* Fisch./Liquorice Root	3,5,2′,4′-tetrahydroxychalcone	N	4 mg/kg	SUA and the content of Hepatic XOD were significantly reduced	It is mainly involved in inhibiting XOD activity to inhibit UA production and down-regulating the protein expression of GLUT9 to inhibit UA re-absorption	[59]
*Morus alba* L./Mori Cortex	Flavonoids of Mori Cortex	H	1 mg/kg	URAT1 was significantly decreased, the content of OAT1 mRNA was significantly increased	It may be related to the down-regulation of URAT1 and the up-regulation OAT1 to promote renal excretion of UA	[60]
*Morus alba* L./Mulberry Leaf	Morusin	J	40 and 80 mg/kg	Increased urinary UA/creatinine ratio and resulting in reduction of SUA level	Down-regulated of renal mGLUT9 and mURAT1, and increased urate secretion via up-regulating of renal mOAT1 to promote renal excretion of UA	[44]
*Morus alba* L./Mulberry Leaf	Mulberry leaf flavonoids	H	50, 100, and 200 mg/kg	SUA and urea nitrogen were effectively lowered, XOD was inhibited	It is related to inhibiting the activity of XOD to inhibit UA production	[61]
*Morus alba* L./Mulberry	Mulberry flavonoids	H	200 mg/kg	SUA were effectively lower	It is related to inhibiting the activity of XOD to inhibit UA production	[56]
*Crataegus pinnatifida* Bge./Hawthorn	Flavonoids of hawthorn leaves	J	3, 6, and 9 mg/kg	SUA was effectively lowered, XOD was inhibited	It is related to inhibiting the activity of XOD to inhibit UA production	[62]
*Sophora japonica* L./Sophora Japonica	Rutin	D	50 and 100 mg/kg	Significantly decreased SUA, BUN, and Scr, and increased urine creatinine excretion	It is related to promoting renal excretion of UA by down-regulating mRNA and protein levels of URAT1 and GLUT9, and up-regulating mRNA and protein levels of OAT1	[63,64]
*Hippophae rhamnoides* L./Seabuckthorn	Isorhamnetin	M	300 mg/kg	Significantly reduced plasma and hepatic UA level, also decreased hepatic XOD activity	It is related to inhibiting the activity of XOD to inhibit UA production	[65]

**Table 3:** In vitro experiment and mechanism of flavonoids bioactive components from plant-based functional foods on hyperuricemia.

Source	Bioactive Compound	Model	Dose	IC_50_	Effects	Mechanisms	Ref.
*Pueraria lobata* (Willd.) Ohwi/Pueraria	Puerarin	Human renal proximal tubular epithelial cells (HK2 cells)	100 mg/L	16.48 µM	Effectively promoted ABCG2 protein expression in HK2 cells	It is related to up-regulating of ABCG2 to promote renal excretion of UA	[66]
*Citrus aurantium* L./Fructus Aurantii	Hesperetin	XOD inhibitor screening model in vitro	20 µM	16.48 µM	Significantly inhibited XOD activity	This is related to inhibit XOD to inhibit UA production	[67]
*Citrus aurantium* L./Fructus Aurantii	Nobiletin	XOD inhibitor screening model in vitro	20 µM	16.48 µM	Significantly inhibited XOD activity	This is related to inhibit XOD to inhibit UA production	[67]
*Citrus reticulata* Blanco/Citrus	Acacatechin	XOD model in vitro	100 µg/mL	27 ± 1.16 µg/mL	Significantly inhibited XOD activity	It showed competitive type of XOD inhibition to inhibit UA production	[68]
*Citrus reticulata* Blanco/Citrus	Glycitein	XOD model in vitro	100 µg/mL	12 ± 0.86 µg/mL	Significantly inhibited XOD activity	It showed competitive type of XOD inhibition to inhibit UA production	[68]
*Citrus reticulata* Blanco/Citrus	Myricetin	XOD model in vitro	100 µg/mL	26 ± 0.72 µg/mL	Significantly inhibited XOD activity	It showed competitive type of XOD inhibition to inhibit UA production	[68]
*Carthamus tinctorius* L./Carthami Flos	Galuteolin	XOD inhibitor screening model in vitro	100 µg/mL	12 ± 0.86 µg/mL	Significantly inhibited XOD activity	This is related to inhibiting XOD to inhibit UA production	[68]
*Citrus reticulata* Blanco/Citrus	Naringenin	XOD model in vitro	100 µg/mL	22 ± 0.64 µg/mL	Significantly inhibited XOD activity	It showed competitive type of XOD inhibition to inhibit UA production	[68]
*Carthamus tinctorius* L./Carthami Flos	Kaemperfol	XOD inhibitor screening model in vitro	100 µg/mL	12 ± 0.86 µg/mL	Significantly inhibited XOD activity	This is related to inhibiting XOD to inhibit UA production	[69]

**Table 4:** Experiment and mechanism of phenolic acid bioactive components from plant-based functional foods on hyperuricemia.

Source	Bioactive Compound	Model	Dose	Effects	Mechanisms	Ref.
*Cichorium intybus* L./Chicory	Chlorogenic acid	R	50 and 150 mg/kg	SUA level significantly was reduced, XOD and ADA levels showed different degrees of inhibition	This is related to promoting UA excretion by down-regulating the expression of mURAT1 and inhibiting XOD and ADA	[76]
*Glycyrrhiza uralensis* Fisch/Liquorice Root	Protocatechuic acid	F	10 mg/kg	SUA level significantly reduced, fractional excretion of uric acid was increased	This is related to down-regulation the transport activity of URAT1 by inhibiting UA re-absorption	[55]
*Coix lachryma-jobi* L./Adlay	Vanillic acid	B	166 mg/kg	SUA level significantly reduced, XOD was inhibited	This is related to inhibiting the activity of XOD	[74]
*Coix lachryma-jobi* L./Adlay	Ferulic acid	B	166 mg/kg	SUA level significantly reduced, XOD was inhibited	This is related to inhibiting the activity of XOD	[74]

**Table 5:** Experiment and mechanism of alkaloids bioactive components from plant-based functional foods on hypouricemia.

Source	Bioactive Compound	Model	Dose	Effects	Mechanisms	Ref.
*Evodia rutaecarpa* (Juss.) Benth./Euodiae Fructus	Evodiamine	Q	8 mg/kg	SUA and XOD could be significantly reduced	This is related to inhibiting the activity of XOD to inhibit of UA production	[83]
*Lycium barbarum* L./Lycii Fructus	Betaine	D	10, 20, and 40 mg/kg	SUA, BUN, and Scr levels significantly reduced, fractional excretion of uric acid was increased	This is related to down-regulating mRNA and protein levels of URAT1 and GLUT9, and up-regulating mRNA and protein levels of OAT1 to promote uric acid excretion	[84,85]

**Table 6:** Experiment and mechanism of other bioactive components from plant-based functional foods on Hyperuricemia.

Source	Bioactive Compound	Model	Dose	Effects	Mechanisms	Ref.
*Camellia sinensis* L./Green tea	Green tea polyphenols	P	600 mg/kg	Decreased SUA and increased excretion of exceeding UA significantly	It can inhibit XOD activities	[101]
*Plantago asiatica* L./Plantaginis Semen	Acteoside	D	200 mg/kg	UA and creatinine levels were obviously reduced and the activity of hepatic XOD was inhibited. Furthermore, the mRNA expression of URAT1 and GLUT9 were obviously down-regulated	The mechanism of lowering SUA level can inhibit XOD activity and down-regulate the mRNA expression of URAT1 and GLUT9	[99,102]
*Morus alba* L./Mori Cortex	Mulberroside A	C	10, 20 and 40 mg/kg	Decreased SUA level and increased urinary UA excretion and fractional excretion of UA. Furthermore, down-regulated mRNA and protein levels of mGLUT9 and mURAT1, and upregulated mRNA and protein levels of mOAT1, mOCT1, mOCT2, mOCTN1, and mOCTN2	Hypouricemic effect is achieved by down-regulating mRNA and protein levels of mGLUT9 and mURAT1, and upregulating mRNA and protein levels of mOAT1 to promote UA excretion	[103]
*Cichorium intybus* L./Chicory	Esculinhydrate	M	50 and 150 mg/kg	SUA level significantly increased, XOD and ADA levels showed different degrees of inhibition	This is related to down-regulation the expression of mURAT1 to promote UA excretion	[76]
*Gardenia jasminoides* Ellis/Cape Jasmine	Geniposide	B	50 and 100 mg/kg	The protein and mRNA expression of URAT1 and GLUT9 and serum UA significantly decreased, while 24 h urinary, the protein and mRNA expression of OAT1 were significantly increased	Down-regulated URAT1 and GLUT9, and up-regulated OAT1 to promote UA excretion	[98]
*Mangifera indica* L./Mango	Mangiferin	B	6 mg/kg	SUA and the protein expression of URAT1, and GLUT9 were significantly decreased, while 24 h urinary creatinine, the expression of mABCG2 were significantly increased	This is related to down-regulation the protein expression of URAT1, GLUT9 and up-regulation the expression of ABCG2 to promote UA excretion	[104,105]
*Mangifera indica* L./Mango	Norathyriol	O	4 mg/kg	Decreased SUA and markedly increased the fractional excretion of UA	The mechanism of lowering SUA can inhibit XOD activity and up-regulated OAT1.	[106]
*Curcuma longa* L./Turmeric	Curcumin	G	20 and 40 mg/kg	Decreased SUA markedly increased	The mechanism of lowering SUA can inhibit XOD activity	[107,108]

## References

[1] Wang Y.N., Zhao M., Xin Y., Liu J.J., Wang M., Zhao C.J. (2016). ^1^H-NMR and MS based metabolomics study of the therapeutic effect of Cortex Fraxini on hyperuricemic rats. J. Ethnopharmacol..

[2] Zhang Y.L., Su H., Zhang J., Kong J. (2019). The effects of ginsenosides and anserine on the up-regulation of renal aquaporins 1–4 in hyperuricemic mice. Am. J. Chin. Med..

[3] Corey-Bloom J., Haque A., Aboufadel S., Snell C., Fischer R.S., Granger S.W., Granger D.A., Thomas E.A. (2020). Uric acid as a potential peripheral biomarker for disease features in huntington’s patients. Front. Neurosci..

[4] Brook R.A., Forsythe A., Smeeding J.E., Lawrence Edwards N. (2010). Chronic gout: Epidemiology, disease progression, treatment and disease burden. Curr. Med. Res. Opin..

[5] Xia Y., Wu Q.J., Wang H.Y., Zhang S., Jiang Y.T., Gong T.T., Xu X., Chang Q., Niu K.J., Zhao Y. (2019). Global, regional and national burden of gout, 1990–2017: A systematic analysis of the Global Burden of Disease Study. Rheumatology.

[6] Michael C.X., Yokose C., Rai S.K., Pillinger M.H., Choi H.K. (2019). Contemporary prevalence of gout and hyperuricemia in the united states and decadal trends: The national health and nutrition examination survey 2007–2016. Arthritis Rheumatol..

[7] Mu Z.P., Wang W., Wang J., Lv W.S., Chen Y., Wang F., Yu X.L., Wang Y.G., Cheng B.F., Wang Z.C. (2019). Predictors of poor response to urate-lowering therapy in patients with gout and hyperuricemia: A post-hoc analysis of a multicenter randomized trial. Clin. Rheumatol..

[8] Chen S., Guo X.F., Dong S.Y., Yu S.S., Chen Y.T., Zhang N.J., Sun Y.X. (2017). Association between the hypertriglyceridemic waist phenotype and hyperuricemia: A cross-sectional study. Clin. Rheumatol..

[9] Ragab G., Elshahaly M., Bardin T. (2017). Gout: An old disease in new perspective—A review. J. Adv. Res..

[10] Li C.G., Hsieh M.C., Chang S.J. (2013). Metabolic syndrome, diabetes, and hyperuricemia. Curr. Opin. Rheumatol..

[11] Petreski T., Ekart R., Hojs R., Bevc S. (2019). Asymptomatic hyperuricemia and cardiovascular mortality in patients with chronic kidney disease who progress to hemodialysis. Int. Urol. Nephrol..

[12] Liu F., Du G.L., Song N., Ma Y.T., Li X.M., Gao X.M., Yang Y.N. (2019). Hyperuricemia and its association with adiposity and dyslipidemia in Northwest China: Results from cardiovascular risk survey in Xinjiang (CRS 2008–2012). Lipids Health Dis..

[13] Jeon H.J., Oh J., Shin D.H. (2019). Urate-lowering agents for asymptomatic hyperuricemia in stage 3–4 chronic kidney disease: Controversial role of kidney function. PLoS ONE.

[14] Guo L.F., Chen X., Lei S.S., Li B., Zhang N.Y., Ge H.Z., Yang K., Lv G.Y., Chen S.H. (2020). Effects and mechanisms of Dendrobium officinalis six nostrum for treatment of hyperuricemia with hyperlipidemia. Evid. Based Complement. Altern. Med..

[15] Maiuolo J., Oppedisano F., Gratteri S., Muscoli C., Mollace V. (2016). Regulation of uric acid metabolism and excretion. Int. J. Cardiol..

[16] Chen C.Y., Lv J.M., Yao Q. (2016). Hyperuricemia-related diseases and xanthine oxidoreductase (XOR) inhibitors: An overview. Med. Sci. Monit..

[17] Han S., Wei R.H., Han D., Zhu J.X., Luo W.Z., Ao W., Zhong G.Y. (2020). Hypouricemic effects of extracts from Urtica *hyperborea* Jacq. ex Wedd. in hyperuricemia mice through XOD, URAT1, and OAT1. BioMed Res. Int..

[18] Liu X.R., Huang S.S., Xu W.D., Zhou A.J., Li H., Zhang R., Liu Y., Yang Y., Jia H. (2018). Association of dietary patterns and hyperuricemia: A cross-sectional study of the Yi ethnic group in China. Food Nutr. Res..

[19] Li R.R., Yu K., Li C.W. (2018). Dietary factors and risk of gout and hyperuricemia: A meta-analysis and systematic review. Asia. Pac. J. Clin. Nutr..

[20] Büsing F., Hägele F.A., Nas A., Döbert L.V., Fricker A., Dörner E., Podlesny D., Aschoff J., Pöhnl T., Schweiggert R. (2018). High intake of orange juice and cola differently affects metabolic risk in healthy subjects. Clin. Nutr..

[21] Perez-Ruiz F., Dalbeth N., Bardin T. (2015). A review of uric acid, crystal deposition disease, and gout. Adv. Ther..

[22] Ristic B., Sikder M.O.F., Bhutia Y.D., Ganapathy V. (2020). Pharmacologic inducers of the uric acid exporter ABCG2 as potential drugs for treatment of gouty arthritis. Asian J. Pharm. Sci..

[23] Xu L.Q., Shi Y.F., Zhuang S.G., Liu N. (2017). Recent advances on uric acid transporters. Oncotarget.

[24] Tan P.K., Liu S., Gunic E., Miner J.N. (2017). Discovery and characterization of verinurad, a potent and specific inhibitor of URAT1 for the treatment of hyperuricemia and gout. Sci. Rep..

[25] DeBosch B.J., Kluth O., Fujiwara H., Schürmann A., Moley K. (2014). Early-onset metabolic syndrome in mice lacking the intestinal uric acid transporter SLC2A9. Nat. Commun..

[26] Liu N.X., Wang Y., Yang M.F., Bian W.X., Zeng L., Yin S.G., Xiong Z.Q., Hu Y., Wang S.Y., Meng B.L. (2018). New rice-derived short peptide potently alleviated hyperuricemia induced by potassium oxonate in rats. J. Agric. Food Chem..

[27] Gliozzi M., Malara N., Muscoli S., Mollace V. (2016). The treatment of hyperuricemia. Int. J. Cardiol..

[28] Pinela J., Carocho M., Dias M.I., Caleja C., Barros L., Ferreira I.C.F.R. (2016). Wild plant-based functional foods, drugs, and nutraceuticals. Wild Plants Mushrooms Nuts..

[29] Kumar A., Mosa K.A., Ji L.Y., Kage U., Dhokane D., Karre S., Madalageri D., Pathania N. (2018). Metabolomics assisted biotechnological interventions for developing plant-based functional foods and nutraceuticals. Crit. Rev. Food. Sci. Nutr..

[30] Ji M.Y., Bo A., Yang M., Xu J.F., Jiang L.L., Zhou B.C., Li M.H. (2020). The pharmacological effects and health benefits of *Platycodon grandiflorus*-A medicine food homology species. Foods.

[31] Gong X., Ji M.Y., Xu J.P., Zhang C.H., Li M.H. (2019). Hypoglycemic effects of bioactive ingredients from medicine food homology and medicinal health food species used in China. Crit. Rev. Food Sci. Nutr..

[32] Badimon L., Vilahur G., Padro T. (2010). Nutraceuticals and atherosclerosis: Human trials. Cardiovasc. Ther..

[33] Mehmood A., Zhao L., Wang C.T., Nadeem M., Raza A., Ali N., Shah A.A. (2019). Management of hyperuricemia through dietary polyphenols as a natural medicament: A comprehensive review. Crit. Rev. Food Sci..

[34] Arimboor R., Arumughan C. (2012). Effect of polymerization on antioxidant and xanthine oxidase inhibitory potential of sea buckthorn (*H. rhamnoides*) proanthocyanidins. J. Food Sci..

[35] Ji M.Y., Gong X., Li X., Wang C.C., Li M.H. (2020). Advanced research on the antioxidant activity and mechanism of polyphenols from *Hippophae* Species—A Review. Molecules.

[36] Chen L., Li M., Wu J.L., Li J.X., Ma Z.C. (2019). Effect of lemon water soluble extract on hyperuricemia in mouse model. Food Funct..

[37] Kapinova A., Stefanicka P., Kubatka P., Zubor P., Uramova S., Kello M., Mojzis J., Blahutova D., Qaradakhi T., Zulli A. (2017). Are plant-based functional foods better choice against cancer than single phytochemicals? A critical review of current breast cancer research. Biomed. Pharmacother..

[38] Lin S.Y., Zhang G.W., Liao Y.J., Pan J.H. (2015). Inhibition of chrysin on xanthine oxidase activity and its inhibition mechanism. Int. J. Biol. Macromol..

[39] Patra J.C., Chua B.H. (2011). Artificial neural network-based drug design for diabetes mellitus using flavonoids. J. Comput. Chem..

[40] Lin S.Y., Zhang G.W., Liao Y.J., Pan J.H. (2015). Dietary flavonoids as xanthine oxidase inhibitors: Structure-Affinity and Structure-Activity relationships. J. Agric. Food Chem..

[41] Lin C.M., Chen C.S., Chen C.T., Liang Y.C., Lin J.K. (2002). Molecular modeling of flavonoids that inhibits xanthine oxidase. Biochem. Biophys. Res. Commun..

[42] Cheng L.C., Murugaiyah V., Chan K.L. (2015). Flavonoids and phenylethanoid glycosides from Lippia nodiflora as promising antihyperuricemic agents and elucidation of their mechanism of action. J. Ethnopharmacol..

[43] Montoro P., Braca A., Pizza C., De Tommasi N. (2005). Structure-antioxidant activity relationships of flavonoids isolated from different plant species. Food Chem..

[44] Wang C.P., Wang X., Zhang X., Shi Y.W., Liu L., Kong L.D. (2010). Morin improves urate excretion and kidney function through regulation of renal organic ion transporters in hyperuricemic mice. J. Pharm. Pharm. Sci..

[45] Xing Z.H., Ma Y.C., Li X.P., Zhang B., Zhang M.D., Wan S.M., Yang X., Yang T.F., Jiang J.W., Bao R. (2017). Research progress of puerarin and its derivatives on anti-inflammatory and anti-gout activities. China J. Chin. Mater. Med..

[46] Xie K.L., Li Z.H., Dong X.Z., Gong M.X. (2019). Research progress of quercetin on inhibiting the activity of xanthine oxidase. Lishizhen Med. Mater. Med. Res..

[47] Shi Y.L., Williamson G. (2016). Quercetin lowers plasma uric acid in pre-hyperuricaemic males: A randomised, double-blinded, placebo-controlled, cross-over trial. Brit. J. Nutr..

[48] Zhang C., Wang R., Zhang G.W., Gong D.M. (2018). Mechanistic insights into the inhibition of quercetin on xanthine oxidase. Int. J. Biol. Macromol..

[49] Zhang Z.C., Su G.H., Luo C.L., Pang Y.L., Wang L., Li X., Zhang J.L. (2015). Effects of anthocyanins from purple sweet potato (Ipomoea batatas L. cultivar Eshu No. 8) on the serum uric acid level and xanthine oxidase activity in hyperuricemic mice. Food Funct..

[50] Qian X.Y., Wang X., Luo J., Liu Y., Pang J., Zhang H.Y., Xu Z.L., Xie J.W., Jiang X.W., Ling W. (2019). Hypouricemic and nephroprotective roles of anthocyanins in hyperuricemic mice. Food Funct..

[51] Meehmood A., Zhao L., Chengtao W., Hossen I., Raka R.N., Zhang H. (2019). Stevia residue extract increases intestinal uric acid excretion via interacting with intestinal urate transporters in hyperuricemic mice. Food Funct..

[52] Miao M.X., Wang X., Lu Y., Wang X. (2016). Mechanism Study on effects of apigenin on reducing uric acid and renal protection in oteracil potassium-induced hyperuricemia mice. China Pharm..

[53] Hao Y., Jiao A.N., Yu M., Gao J.Z., He X., Zhang M.H., Jiao L.Q., Zhang J. (2019). Activity screening of thirty flavonoids on the inhibition of xanthine oxidase. Chin. Tradit. Pat. Med..

[54] Zhao R., Chen D., Wu H.L. (2017). Pu-erh ripened tea resists to hyperuricemia through xanthine oxidase and renal urate transporters in hyperuricemic mice. J. Funct. Foods..

[55] Wang Z., Ci X.Y., Cui T., Wei Z.H., Zhang H.B., Liu R., Li Y.Z., Yi X.L., Zhang T.J., Gu Y. (2019). Effects of Chinese herb ingredients with different properties on OAT4, URAT1 and serum uric acid level in acute hyperuricemia mice. Chin. Trad. Herb. Drugs.

[56] Wang H.Q., Zhan J., Wang X.B., Zou L. (2015). Research progress in treatment of hyperuricemia with active ingredients of traditional Chinese medicine. Chin. J. Pharmacol. Toxicol..

[57] Wang M., Zhao J., Zhang N., Chen J.H. (2016). Astilbin improves potassium oxonate-induced hyperuricemia and kidney injury through regulating oxidative stress and inflammation response in mice. Biomed. Pharm..

[58] Shi K., Zhang R.T., Shang X.Y., Wang N., Li S., Zhang Z.S. (2014). Effect of puerarin on serum uric acid in hyperuricemic rat. Food Sci. Technol..

[59] Pu J.Y., Niu Y.F., Gao L.H., Lin H., Tu C.X., Li L. (2015). Effects of 3,5,2′,4′-tetrahydroxychalcone on urate excretion in hyperuricemic mice. Chin. Pharmacol. Bull..

[60] Dang Y.X., Liang D.L., Zhou X.X., Qin Y., Gao Y., Li W.M. (2019). Protective effect of Mori Cortex on kidney in rats with hyperlipidemia and hyperuricemia based on molecular docking technique. Chin. Trad. Herb. Drugs.

[61] Zhang H.C., Zhang Y., Lv G.F., Wang E.P., Chen X. (2016). The puerarin impact on the expression of ABCG2 in human renal proximal tubule epithelial cells. SH J. TCM Mar..

[62] Wang K., Wang R.P., Li J., Zhao D., Wang J.Q., Ran X., Qu W.J. (2012). The preventive and therapeutic effects of mulberry leaf flavonoids on adenine induced hyperuricemia and kidney injury in rats. Nat. Prod. Res. Dev..

[63] Zhang Z.G., Yang H. (2012). Effects of total flavone of hawthorn leaf on serum uric acid and vascular endothelial cell function in hyperuricemia rats. Chin. J. Exp. Trad. Med. Formulae.

[64] Liu J.L., Li L.Y., He G.H., Zhang X., Song X.H., Cui G.L., Liao S.Q. (2018). Quality evaluation of Flos Sophorae Immaturus from different habitats by HPLC coupled with chemometrics and anti-oxidant ability. Chin. Trad. Herb. Drugs.

[65] Chen Y.S., Hu Q.H., Zhang X., Zhu Q., Kong L.D. (2013). Beneficial effect of rutin on oxonate-induced hyperuricemia and renal dysfunction in mice. Pharmacology.

[66] Adachi S.I., Kondo S., Sato Y., Yoshizawa F., Yagasaki K. (2019). Anti-hyperuricemic effect of isorhamnetin in cultured hepatocytes and model mice: Structure-activity relationships of methylquercetins as inhibitors of uric acid production. Cytotechnology.

[67] Liu K., Wang W., Guo B.H., Gao H., Liu Y., Liu X.H., Yao H.L., Cheng K. (2016). Chemical evidence for potent xanthine oxidase inhibitory activity of ethyl acetate extract of *Citrus aurantium* L. dried immature fruits. Molecules.

[68] Umamaheswari M., Madeswaran A., Asokkumar K. (2013). Virtual screening analysis and in-vitro xanthine oxidase inhibitory activity of some commercially available flavonoids. Iran. J. Pharm. Res..

[69] Yu S.H., Song H.P., Gao W., Zhang H. (2017). Study on the inhibitory activity of flavonoids in *Carthami Flos* on xanthine oxidase. Chin. J. Ethnomed. Ethnopharm..

[70] González-Castejón M., Rodriguez-Casado A. (2011). Dietary phytochemicals and their potential effects on obesity: A review. Pharmacol. Res..

[71] Irondi E.A., Agboola S.O., Oboh G., Boligon A.A., Athayde M.L., Shode F.O. (2016). Guava leaves polyphenolics-rich extract inhibits vital enzymes implicated in gout and hypertension in vitro. J. Intercult. Ethnopharm..

[72] Zhu C.S., Zhang B., Lin Z.J., Bai Y.F. (2018). Pharmacodynamics authentication research on uric acid-lowering active ingredients of *Cichorium intybus* L.. China J. Trad. Chin. Med. Pharm..

[73] Zhu C.S., Lin Z.J., Zhang B., Wang H.B., Wang X.J., Niu H.J., Wang Y., Niu A.Z. (2015). Spectrum-effect relationships on uric acid lowering effect of *Cichorium intybus*. Chin. Trad. Herb. Drugs.

[74] Zhao M.M., Zhu D.S., Sun-Waterhouse D.X., Su G.W., Lin L.Z., Wang X., Dong Y. (2014). In vitro and in vivo studies on adlay-derived seed extracts: Phenolic profiles, antioxidant activities, serum uric acid suppression, and xanthine oxidase inhibitory effects. J. Agric. Food Chem..

[75] Wan Y., Wang F., Zou B., Shen Y.F., Li Y.Z., Zhang A.X., Fu G.M. (2019). Molecular mechanism underlying the ability of caffeic acid to decrease uric acid levels in hyperuricemia rats. J. Funct. Foods..

[76] Zhu C.S., Lin Z.J., Zhang B., Bai Y.F. (2017). Uric acid-lowering active ingredients and mechanism of *Cichorium intybus*. Chin. Trad. Herb. Drugs.

[77] Qiu S., Sun H., Zhang A.H., Xu H.Y., Yan G.L., Han Y., Wang X.J. (2014). Natural alkaloids: Basic aspects, biological roles, and future perspectives. Chin. J. Nat. Med..

[78] Zou L., Feng F.Q. (2019). Research progress of uric acid-lowering bioactive compounds in food and their mechanisms. Sci. Technol. Food Ind..

[79] Sang M.M., Du G.Y., Hao J., Wang L.L., Liu E.W., Zhang Y., Wang T., Gao X.M., Han L. (2017). Modeling and optimizing inhibitory activities of Nelumbinis folium extract on xanthine oxidase using response surface methodology. J. Pharm. Biomed..

[80] Wang M.X., Liu Y.L., Yang Y., Zhang D.M., Kong L.D. (2015). Nuciferine restores potassium oxonate-induced hyperuricemia and kidney inflammation in mice. Eur. J. Pharmacol..

[81] Tao Z.Y., Cheng Y., Tang Y., Tan Y.M., Li J. (2014). Effect of evodiamine on the animal model of Hyperuricemia. Pharm. Clin. Chin. Mater. Med..

[82] Song Y., Li J., Cheng Y., Lin Z., He B.Y., Wang C.Y. (2015). Lowering effect of evodiamine dispersible tablets on uric acid in chickens. Chin. J. New Drugs.

[83] Hu M., Liu J.W., Song Y., Zeng N. (2014). Effect and mechanism study of evodiamine on hyperuricemia model quail. Pharmacol. Clin. Chin. Mater. Med..

[84] Tan L., Ji T., Cao J.Y., Hu F.Z. (2014). Determination of betaine contents in Fructus Lycii from different origins by dual wavelength TLC scanning. Nat. Prod. Res. Dev..

[85] Liu Y.L., Pan Y., Wang X., Fan C.Y., Zhu Q., Li J.M., Wang S.J., Kong L.D. (2013). Betaine Reduces Serum Uric Acid Levels and Improves Kidney Function in Hyperuricemic Mice. Planta Med..

[86] Li P., Song Z.B., Chen M.M., Song J., Cui H.X. (2018). Research progress of therapeutic drug of hyperuricemia and its action target. China Mod. Med..

[87] Gong L.X., Chi J.W., Wang J., Ren Y.Q., Sun B.G. (2019). Research progress on main functional component and action mechanism of *Dioscorea opposita*. Sci. Technol. Food Ind..

[88] Su J.X., Wei Y.H., Liu M.L., Liu T.X., Li J.H., Ji Y.C., Liang J.P. (2014). Anti-hyperuricemic and nephroprotective effects of *Rhizoma Dioscoreae* septemlobae extracts and its main component dioscin via regulation of mOAT1, mURAT1 and mOCT2 in hypertensive mice. Arch. Pharm. Res..

[89] Shi S., Wang N., Shang X.Y., Zhang R.T., Li S., Zhang Z.S. (2014). Effect of gypenoside on serum uric acid of hyperuricemic rats. Nat. Prod. Res. Dev..

[90] Pang M.X., Fang Y.Y., Chen S.H., Zhu X.X., Shan C.W., Su J., Yu J.J., Li B., Yang Y., Chen B. (2017). Gypenosides inhibits xanthine oxidoreductase and ameliorates urate excretion in hyperuricemic rats induced by high cholesterol and high fat food (Lipid Emulsion). Med. Sci. Monit..

[91] Meng F.C., Li Q., Qi Y.M., He C.W., Wang C.M., Zhang Q.W. (2018). Characterization and immunoregulatory activity of two polysaccharides from the root of *Ilex asprella*. Carbohydr. Polym..

[92] Liu M., Li S.S., Wang X.X., Zhu Y.F., Zhang J.J., Liu H., Jia L. (2018). Characterization, anti-oxidation and anti-inflammation of polysaccharides by *Hypsizygus marmoreus* against LPS-induced toxicity on lung. Int. J. Biol. Macromol..

[93] Yang Q.X., Wang Q.L., Deng W.W., Sun C.Y., Wei Q.Y., Adu-Frimpong M., Shi J.X., Yu J.N., Xu X.M. (2019). Anti-hyperuricemic and anti-gouty arthritis activities of polysaccharide purified from *Lonicera japonica* in model rats. Int. J. Biol. Macromol..

[94] Deng L.J., Yan J.X., Wang P., Zhou Y., Wu X.A. (2019). Effects of pachman on the expression of renal tubular transporters rURAT1, rOAT1 and rOCT2 of the rats with hyperuricemia. West. J. Tradit. Chin. Med..

[95] Lanaspa M.A., Ishimoto T., Cicerchi C., Tamura Y., Roncal-Jimenez C.A., Chen W., Johnson R.J. (2014). Endogenous fructose production and fructokinase activation mediate renal injury in diabetic nephropathy. J. Am. Soc. Nephrol..

[96] Lecoultre V., Egli L., Theytaz F., Despland C., Schneiter P., Tappy L. (2013). Fructose-induced hyperuricemia is associated with a decreased renal uric acid excretion in humans. Diabetes Care.

[97] Cirillo P., Gersch M.S., Mu W., Scherer P.M., Kim K.M., Gesualdo L., Henderson G.N., Johnson R.J., Sautin Y.Y. (2009). Ketohexokinase-dependent metabolism of fructose induces proinflammatory mediators in proximal tubular cells. J. Am. Soc. Nephrol..

[98] Zhou J., Sun C., Li F. (2018). Research advances in mechanism of active components of traditional Chinese medicine for reducing uric acid. Chin. Pharmacol. Bull..

[99] Zeng J.X., Xu B.B., Wang J., Bi Y., Wang X.Y., Zhong G.Y., Ren G., Zhu J.X., Li M., Zhu Y.Y. (2016). Hypouricemic effects of acteoside and isoacteoside from *Plantaginis Semen* on mice with acute hyperuricemia and their possible mechanisms. Chin. Tradit. Pat. Med..

[100] Moriwaki Y.J., Okuda C., Yamamoto A., Ka T., Tsutsumi Z., Takahashi S., Yamamoto T., Kitadate K., Wakame K. (2011). Effects of oligonol, an oligomerized polyphenol formulated from lychee fruit, on serum concentration and urinary excretion of uric acid. J. Func. Foods.

[101] Nugraheni P.W., Rahmawati F., Mahdi C., Prasetyawan S. (2017). Green tea extract (*Camellia sinensis* L.) effects on uric acid levels on hyperuricemia rats (*Rattus norvegicus*). J. Pure App. Chem. Res..

[102] Huang C.G., Shang Y.J., Zhang J., Zhang J.R., Li W.J., Jiao B.H. (2008). Hypouricemic effects of phenylpropanoid glycosides acteoside of scrophularia ningpoensis on serum uric acid levels in potassium oxonate-pretreated mice. Am. J. Chin. Med..

[103] Wang C.P., Wang Y., Wang X., Zhang X., Ye J.F., Hu L.S., Kong L.D. (2011). Mulberroside A possesses potent uricosuric and nephroprotective effects in hyperuricemic mice. Planta Med..

[104] Xu X.W., Niu Y.F., Gao L.H., Li L., Lin H. (2018). Analysis of hypouricemic mechanism of mangiferin based on intestinal urate transporter ABCG2. Chin. J. Exp. Tradit. Med. Formulae.

[105] Yang H., Gao L.H., Niu Y.F., Zhou Y.F., Lin H., Jiang J., Kong X.F., Liu X., Li L. (2015). Mangiferin inhibits renal urate reabsorption by modulating urate transporters in experimental hyperuricemia. Biol. Pharm. Bull..

[106] Lin H., Tu C.X., Niu Y.F., Li F.S., Yuan L.X., Li N., Xu A.P., Gao L.H., Li L. (2019). Dual actions of norathyriol as a new candidate hypouricaemic agent: Uricosuric effects and xanthine oxidase inhibition. Eur. J. Pharmacol..

[107] Chen Y.E., Li C.T., Duan S.N., Yuan X., Liang J., Hou S.Z. (2019). Curcumin attenuates potassium oxonate-induced hyperuricemia and kidney inflammation in mice. Biomed. Pharmacother..

[108] Ao G.Z., Zhou M.Z., Li Y.Y., Li S.N., Wang H.N., Wan Q.W., Li H.Q., Hu Q.H. (2017). Discovery of novel curcumin derivatives targeting xanthine oxidase and urate transporter 1 as anti-hyperuricemic agents. Bioorg. Med. Chem..

[109] Li X.Z., Zheng L.L., Ai B.L., Zheng X.Y., Yang Y., Yang J.S., Sheng Z.W. (2020). The inhibitory kinetics and mechanism of xanthine oxidase by screened polyphenols. Food Res. Dev..

[110] Lin L., Yang Q., Zhao K., Zhao M. (2018). Identification of the free phenolic profile of Adlay bran by UPLC-QTOF-MS/MS and inhibitory mechanisms of phenolic acids against xanthine oxidase. Food Chem..

[111] Lin L.C., Pai Y.F., Tsai T.H. (2015). Isolation of luteolin and luteolin-7-*O*-glucoside from *Dendranthema morifolium* Ramat Tzvel and their pharmacokinetics in rats. J. Agric. Food Chem..

[112] Yan J., Zhang G., Hu Y., Ma Y. (2013). Effect of luteolin on xanthine oxidase: Inhibition kinetics and interaction mechanism merging with docking simulation. Food Chem..

[113] Pu Z.Q., Wang Q.L., Xu X.M., Yu J.N. (2017). Separation, purification of galangin and its effect on reducing uric acid. J. Jiangsu Univ..

[114] Zhang C., Zhang G.W., Pan J.H., Gong D.M. (2016). Galangin competitively inhibits xanthine oxidase by a ping-pong mechanism. Food Res. Int..

[115] Komazawa H., Yamaguchi H., Hidaka K., Ogura J., Kobayashi M., Iseki K. (2013). Renal Uptake of substrates for organic anion transporters Oat1 and Oat3 and organic cation transporters Oct1 and Oct2 is altered in rats with adenine-induced chronic renal failure. J. Pharm. Sci..

[116] Nakanishi T., Fukushi A., Sato M., Yoshifuji M., Gose T., Shirasaka Y., Ohe K., Kobayashi M., Kawai K., Tamai I. (2011). Functional characterization of apical transporters expressed in rat proximal tubular cells (PTCs) in primary culture. Mol. Pharm..

[117] Tai L.L., Liu Z.H., Sun M.H., Xie Q.J., Cai X.Q., Wang Y., Dong X., Xu Y. (2020). Anti-hyperuricemic effects of three theaflavins isolated from black tea in hyperuricemic mice. J. Funct. Foods..

[118] Yin Y.C., Ma C.C., Wu J., Yu S.L., Guo X.Z., Hou L.A., You T.T., Wang D.C., Li H.L., Xu T. (2018). Association of SLC22A12 and SLC2A9 genetic polymorphisms with hypouricemia in Ningxia populatio. Basic Clin. Med..

[119] Liu D.P. (2016). Hypouricemia. Chin. J. Cardiovasc. Med..

[120] Dong S.T., Chen Y., Gao Q.Y. (2020). Research progress on bioactive compounds and function of sea buckthorn berry. Chin. Brew..

[121] Pei J.B., Li X.Y., Wang J.H. (2011). Study on variation of sugar, acid, vitamin C and pigments contents during fruit development of blueberries. J. Northeast Agric. Univ..

[122] Mandal A.K., Mount D.B. (2015). The molecular physiology of uric acid homeostasis. Annu. Rev. Physiol..

[123] Stiburkova B., Pavelcova K., Pavlikova M., Ješina P., Pavelka K. (2019). The impact of dysfunctional variants of ABCG2 on hyperuricemia and gout in pediatric-onset patients. Arthritis Res. Ther..

[124] Nakayama A., Matsuo H., Nakaoka H., Nakamura T., Nakashima H., Takada Y., Oikawa Y., Takada T., Sakiyama1 M., Shimizu1 S. (2014). Common dysfunctional variants of ABCG2 have stronger impact on hyperuricemia progression than typical environmental risk factors. Sci. Rep..

[125] Matsuo H., Yamamoto K., Nakaoka H., Nakayama A., Sakiyama M., Chiba T. (2015). Genome-wide association study of clinically defined gout identifies multiple risk loci and its association with clinical subtypes. Ann. Rheum. Dis..

[126] Morimoto C., Tamura Y., Asakawa S., Kuribayashi-Okuma E., Nemoto Y., Li J.P., Murase T., Nakamura T., Hosoyamada M., Uchida S. (2020). ABCG2 expression and uric acid metabolism of the intestine in hyperuricemia model rat. Nucleosides Nucleotides Nucleic Acids.

[127] Wang Y., Lin Z., Zhang B., Nie A.Z., Bian M. (2017). *Cichorium intybus* L. promotes intestinal uric acid excretion by modulating ABCG2 in experimental hyperuricemia. Nut. Metab..

